# Utilisation of and Attitude towards Traditional and Complementary Medicine among Ebola Survivors in Sierra Leone

**DOI:** 10.3390/medicina55070387

**Published:** 2019-07-18

**Authors:** Peter Bai James, Jon Wardle, Amie Steel, Jon Adams

**Affiliations:** 1 Australian Research Centre in Complementary and Integrative Medicine, Faculty of Health, University of Technology Sydney, Ultimo, Sydney 2007, Australia; 2 Faculty of Pharmaceutical Sciences, College of Medicine and Allied Health Sciences, University of Sierra Leone, 00232 Freetown, Sierra Leone

**Keywords:** Ebola, Ebola survivors, attitude, beliefs, traditional medicine, complementary medicine, Sierra Leone

## Abstract

*Background and objectives:* In addition to conventional healthcare, Ebola survivors are known to seek traditional and complementary healthcare (T&CM) options to meet their healthcare needs. However, little is known about the general beliefs of Ebola survivors regarding T&CM and the impact of these beliefs in influencing their decisions around T&CM use. This study examines Ebola survivors’ attitudes towards T&CM use in Sierra Leone. *Materials and Methods:* We conducted a nationwide quantitative cross-sectional study of 358 Ebola survivors in Sierra Leone between January and August 2018. We used descriptive analysis, chi-square tests and backward stepwise binary logistic regression for data analysis. *Results:* Close to half of the survivors (n = 163, 45.5%) had used T&CM since their discharge from an Ebola treatment centre. Survivors who viewed T&CM as boosting their immune system/resistance were 3.89 times (95%CI: 1.57–9.63, *p* = 0.003) more likely to use T&CM than those who did not view T&CM as boosting their immune system/resistance. Additionally, survivors who viewed T&CM as having fewer side effects than conventional medicine were more likely to use T&CM [OR = 5.03 (95%CI: 1.92–13.19, *p* = 0.001)]. Ebola survivors were more influenced to use T&CM based on their personal experience of the effectiveness of T&CM than by clinical evidence [OR = 13.72 (95%CI: 6.10–30.84, *P* < 0.001)]. Ebola survivors who perceived T&CM as providing them with more control than conventional medicine over their health/body were more likely to use T&CM [OR = 4.15 (95%CI: 1.74–9.89, *p* = 0.001)] as opposed to those who did not perceive T&CM in this way. *Conclusions:* Considering the widespread use of T&CM, an understanding of Ebola survivors’ attitudes/beliefs towards T&CM is useful to healthcare providers and policymakers with regard to public education and practitioner–survivors communication, T&CM regulation and research in Sierra Leone. Ebola survivors appear to turn to T&CM not only for treatment, but also to fill gaps in conventional health care services.

## 1. Introduction

Traditional and complementary medicine (T&CM) can be defined as a set of healthcare modalities (local or imported products and practices) that are historically located outside the dominant model of healthcare [1,2]. In Africa, T&CM may include traditional healthcare products and practices (herbal medicine or products and traditional bone setting) and imported complementary medicine (acupuncture and massage therapy, yoga naturopathy) [3,4,5]. T&CM use in Africa is considered a public health issue primarily due to its widespread use alone or alongside conventional medicine, with most studies showing more than half the population using T&CM [4]. In Sierra Leone, high use of T&CM, especially herbal medicines use, has been observed among pregnant women [6], lactating mothers [7], hypertensive patients [8], infertile women [9], healthcare students [10] and for the treatment of malaria in children [11] and adults [12].

The 2014–2016 Ebola virus disease (EVD) outbreak in West Africa was considered to be the most significant outbreak in history with EVD infecting 28,616 people and resulting in 11,310 deaths [13]. The outbreak also produced the highest number of survivors in the three most affected countries of Sierra Leone, Liberia and Guinea. As of the 12 May 2016, the global estimate of EVD survivors was approximately 10,000 [14]. This number will increase as EVD outbreaks continue in other countries such in the Democratic Republic of Congo (DR Congo) [15]. Survivors of EVD outbreaks experience a myriad of post-ebola physical and psychological complications, which include musculoskeletal pain, ocular and auditory problems, depression, anxiety, fatigue, insomnia, as well as stigma and discrimination from the community [16,17,18]. Ebola survivors are known to seek conventional care to address their myriad health challenges [19,20] and there is evidence of EVD survivors employing local cultural practices (which encompass T&CM) in their management of EVD transmission and prevention [21,22,23]. However, no research to date has evaluated the frequency and reasons for T&CM use among Ebola survivors.

Two dominant sets of interpretations have been proposed to help explain the reasons why many people around the globe use T&CM. The first set of interpretations can loosely be referred to as pull factors, which focused on those features, and factors around T&CM that are attractive to users (pull factors). These pull factors regarding T&CM use that have been identified in the contemporary literature across sub-Saharan Africa suggest that people tend to be attracted to T&CM due to its perceived low cost, availability, congruence with their individual’s cultural and religious beliefs, patient sense of autonomy over their health and their perceived safety and efficacy of T&CM products [4]. The second interpretation relates to people who are dissatisfied with what conventional medicine can offer (push factors). The contemporary literature across sub-Saharan Africa suggests that people seek T&CM due to disenchantment with conventional medicine, the negative attitude of healthcare providers to cultural traditions (including T&CM), drug availability, long distance to and inequity in accessing health facilities as well as long waiting times [4]. In addition, beliefs related to safety, efficacy, holism and the sense of control of one’s health have been identified as possible predictors of T&CM use among different illness groups outside of Africa [24,25,26,27].

The very few studies that have explored beliefs and attitudes towards the use of T&CM in Sierra Leone have been restricted to healthcare students (showing generally positive attitudes towards T&CM amongst this sub-group) [10,28]. Given that unpublished data has suggested Ebola survivors are likely to seek T&CM healthcare in Sierra Leone to help meet their healthcare needs [29], it is important to examine Ebola survivors’ general beliefs regarding T&CM and how these beliefs are linked with their decision to use T&CM. Such information is important, as it will help healthcare providers and policymakers to understand better Ebola survivors’ use and motivations for using T&CM, which will help minimise risks to patient care and invariably maximize patient care outcomes. These studies may also serve to identify ‘push’ and ‘pull’ factors that can help design more responsive health services for Ebola survivors. However, to date, no study in Sierra Leone or across Africa has evaluated Ebola survivors’ attitudes towards the use of T&CM. To fill this significant research gap, our study evaluates Ebola survivors’ attitudes towards the use of T&CM in Sierra Leone drawing upon a nationally representative sample.

## 2. Materials and Methods

### 2.1. Study Design, Setting and Participants

A descriptive nationwide questionnaire survey employing a cross-sectional study design was administered to Ebola survivors who were at least 18 years old and experiencing post-Ebola sequelae in Sierra Leone. The study was undertaken between January and August 2018. Ebola survivors who were unable to accurately provide information or participate in the study due to physical and psychological conditions such as memory loss, hearing loss, high fever and bleeding or those experiencing acute emotional distress that would put the research and other participants at risk were excluded.

### 2.2. Sampling Method

**Figure 1:**
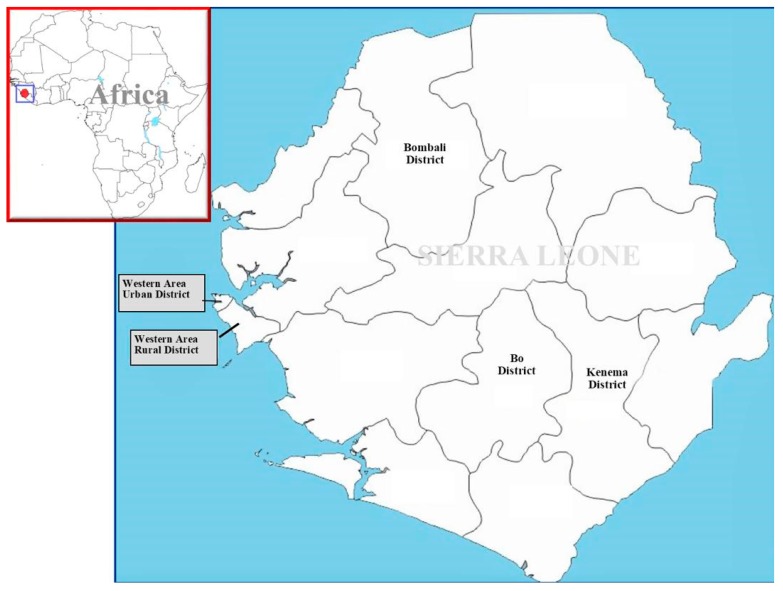
Location of the five sampled districts (Bo District, Kenema District, Bombali District, Western Area Urban District and Western Area Rural District) in Sierra Leone.

We collected data from the four geographic regions of Sierra Leone (Western Area, Northern province, Southern province and Eastern province). We then purposefully selected five districts to cover all four geographic regions of the country. Figure 1 shows the location of the five districts in Sierra Leone. The five districts include western area urban and western area rural districts (both in the Western area), Bo district (Southern province), Kenema district (Eastern province) and Bombali district (Northern province). We chose these five districts based on the epidemiological profile of the total confirmed Ebola cases and because they are host to the highest number of Ebola survivors in Sierra Leone. We then randomly selected Ebola survivors in each of the five districts based on proportional representation using the national list of registered Ebola survivors obtained from the Sierra Leone Association of Ebola survivors (SLAES). Survivors who were randomly selected were approached to participate in the study via telephone. The required sample of 351 Ebola survivors was determined using the formula for cross-sectional study (N = z^2^pq/d^2^), with the perceived prevalence (p) assumed to be 50% since no previous research on T&CM use among Ebola survivors has been conducted so far. To increase the statistical power, we aimed to recruit 400 Ebola survivors.

### 2.3. Use of Traditional, Complementary and Alternative Medicine

Ebola survivors were questioned about their use of T&CM (product and practitioners) since their discharge from an Ebola treatment centre for the management of common post-ebola sequelae. The T&CM products and practices considered in our study included herbal medicine, traditional medicine practice (traditional bone-setting), prayer/faith healing and massage. The T&CM products and practices considered in our study were informed by the results of previous research on T&CM use conducted in Sierra Leone [6,7,8,9,12,28] and across Africa [4].

### 2.4. Attitudes Towards the Use of T&CM among Ebola Survivors

Ebola survivors were asked whether they agree or disagree with the following statements: T&CM has fewer side effects than conventional medicine (CM); T&CM is more natural than CM; T&CM promotes a holistic approach to health; T&CM boosts my immune system/resistance; T&CM gives me more control over my health/body; knowledge about the evidence of T&CM is important to me as a patient; my personal experience of the effectiveness of T&CM is more important than clinical evidence; CAM needs to be tested for safety and side-effects; and T&CM is a better preventative measure than conventional medicine. Survivors were also questioned as to whether they perceived T&CM practitioners as spending a longer time in consultation with and providing more support to their patient than medical doctors. Further, we asked survivors about whether they find it easier to talk to a T&CM practitioner compared to a medical doctor and whether they believe medical doctors should be able to advise patients about T&CM.

### 2.5. Data Collection and Ethical Consideration

Ethical approval was obtained from the University of Technology Sydney Human Research Ethics Committee (UTS-HREC-ETH17-2080, Date of Approval = 19 April 2018) and the Sierra Leone Ethics and Scientific Review Committee (Date of Approval = 17 May 2018). The selected sample of Ebola survivors was approached to take part in the study via telephone during which the scope and rationale, as well as the option to opt out of the study, were explained. Initial verbal consent was obtained via telephone. For those who gave verbal consent, an arrangement was made either to fill the questionnaire or to be interviewed at their homes, courtyard or the regional Ebola survivor office. A participant information sheet that explains the purpose and scope of the study, as well as the option to opt out, was given or read (for illiterate participants) participants before asked to sign or thumbprint the consent form. Survivors signing or thumb printing the consent form was interpreted as their willingness to participate. Survivors who signed or thumbed printed (for illiterate participants) the consent form were then given the questionnaire to fill or to be interviewed. Data were collected from Ebola survivors using self-administered or interviewer-administered (for illiterate participants) formats. Among those who consented to participate in the study, the majority filled the questionnaire in the presence of a data collector or were interviewed by a data collector at the Ebola survivor regional office. Additionally, some Ebola survivors filled the questionnaire alone in their homes at their own time and later sent the filled questionnaires to their regional offices. In addition, some Ebola survivors filled the questionnaire in the presence of a data collector or were interviewed at their homes or the village courtyard. We collected our data between May and August 2018.

### 2.6. Statistical Analysis

Data analysis was conducted using SPSS Statistics version 24. Chi-square or Fischer exact two-tailed tests were used to determine the association between each of the attitude statements and T&CM use. We employed a backward stepwise binary logistic model to determine the attitude statements that are significant predictors of T&CM use. All attitude statements were entered into the model, and a backward stepwise elimination process was conducted until we obtained the most parsimonious model. Ebola survivors’ age, sex, marital status, educational background, financial status, religious affiliation, perceived health status, place of residence, duration (months) since discharged from ETC and presence of chronic disease prior to being infected with Ebola were entered into the model as potential cofounders. The probability value of less than 0.05 was considered statistically significant for all analyses.

## 3. Results

Of the 400 Ebola survivors invited to participate, 376 consented, of which 358 fully completed the questionnaire. Table 1 shows the demographic and health-related characteristics between users and non-users of T&CM among Ebola survivors. More than half Ebola survivors were between the ages of 18–34 years (n = 194, 54.2%) and close to two thirds were females (n = 223, 62.3%). Moreover, close to three-quarters perceived their current health to be fair/poor (n = 262, 73.2%).

Table 2 shows that close to half of the survivors (n = 163, 45.5%) had used T&CM since their discharge from an Ebola treatment centre. Moreover, less than a quarter of survivors (n = 62, 17.3%) used both conventional medicine and T&CM concurrently. Herbal medicine (n = 136, 83.4%) is the most common type of T&CM used among T&CM users.

Table 3 shows the attitudes towards use of T&CM by Ebola survivors. More than two thirds of participants (n = 257, 71.8%) believe that T&CM is ‘natural’. Approximately three-quarters of survivors are of the view that T&CM needs to be tested for safety/side-effects and more than half (n = 213, 59.5%) report knowledge about the evidence of T&CM as important to them. In addition, the majority of Ebola survivors (n = 313, 87.4%) believe medical doctors should be able to advise patients about T&CM. On the other hand, more than half (n = 218, 60.9%) did not perceive T&CM as having fewer side effects than conventional medicine. Furthermore, more than two thirds (n = 250, 69.8%) did not perceive T&CM as promoting a holistic approach to health. In addition, less than one quarter (n = 73, 20.4%) perceived a T&CM practitioner as providing more support to their patients than a medical doctor.

Table 4 outlines the association between T&CM use and individual attitudes of Ebola survivors. Attitude statements such as T&CM is more natural than conventional medicine (*p* < 0.001), T&CM boosts my immune system/resistance (*p* < 0.001), my personal experience of the effectiveness of T&CM is more important than clinical evidence (*p* < 0.001), T&CM gives me more control over my health/body (*p* < 0.001), I find it easier to talk to a T&CM practitioner than a medical doctor (*p* < 0.001) and T&CM has fewer side-effects than conventional medicine (*p* < 0.001) are associated with T&CM use. No statistical difference was observed between T&CM users and non-users for the remaining attitude statements.

Backward stepwise logistic regression (Table 5) found that survivors who agreed that T&CM boosts their immune system/resistance were 3.89 (95%CI: 1.57–9.63, *p* = 0.003) times more likely to use T&CM than those that disagreed. Ebola survivors who agreed with the statement that T&CM has fewer side effects than CM were 5.03 (95%CI: 1.92–13.19, *p* = 0.001) times more likely to use T&CM than those who disagreed. Ebola survivors were 13.72 (95%CI: 6.10–30.84, *p* < 0.001) times more likely to use T&CM if they considered important their personal experience of the effectiveness of T&CM than clinical evidence. In addition, survivors who believe that T&CM gives them more control over their health/body were 4.15 (95%CI: 1.74–9.89, *p* = 0.001) times more likely to be T&CM users as opposed to those who disagreed with this statement.

## 4. Discussion

Our paper presents findings from the first nationwide study to examine Ebola survivors’ attitudes towards the use of T&CM in Sierra Leone. Ebola survivors who use T&CM appear to be of the view that these medicines boost their immune system. Similar reasons have been proffered for using T&CM among those with HIV/AIDS [30] and cancer survivors [31]. The appeal of T&CM to boosting immunity among users in our study may be explained in that, an individual’s health is a function of his/her immune status, which is in line with T&CM philosophies that disease should be managed by challenging the body to heal itself rather than focusing on symptomatic treatment [32].

Additionally, based on the results of this study this group of Ebola survivors may be of the view that the mechanism underlying the pathophysiology of most of their post-ebola sequalae is thought to be immune mediated [33,34,35] and that the use of certain T&CM with immunomodulatory properties will help manage their post-Ebola complications. This is perhaps unsurprising given the infectious nature of the viral disease that caused the acute episode. Although several T&CM approaches are reported to exert immunological changes in preclinical and clinical studies [36], thus far, these studies have employed relatively insensitive and straightforward methodologies that render findings inconclusive. Methodologically robust clinical studies that use newer and more powerful technologies (magnetic-resonance imaging and positron-emission tomography and microarray analyses) are required to provide strong evidence on the immunomodulatory effect of T&CM that might be of use in the management of post-Ebola sequelae among survivors.

Our analyses also shows Ebola survivors who are T&CM users hold the view that T&CM has fewer side effects than conventional medicine mirroring similar findings in the literature on T&CM use among HIV/AIDS patients [37] and individuals with musculoskeletal conditions within [38] and outside [25] of Africa. Moreover, our analysis suggests that Ebola survivors who use T&CM are highly likely to be driven by belief in the notion that T&CM is more natural than conventional medicine. The assumption that T&CM treatments are natural and therefore non-toxic have been found to be associated with people’s decisions to use T&CM in multiple studies in multiple conditions [4,24,39]. T&CM methods and remedies are considered natural and organic as opposed to conventional medicine, which is often thought of as artificial and/or synthetic [24].

Although some T&CM products and practices have been found to be relatively effective and safe, the safety of T&CM in general is still a debatable area—particularly in a nascent condition such as post-Ebola sequelae—as there is insufficient scientific evidence to prove that T&CM is of less risk than conventional medicine [40]. Information regarding the safety of T&CM is mostly derived from community use and are—in most cases—not reliable [41,42]. It is important that well-designed studies provide more examination of such beliefs around safety and T&CM both in the general population and among Ebola survivors.

To further understand T&CM safety, it is also important for researchers to take into consideration the fact that the risk of T&CM needs to be viewed through a wider lens of missed opportunity for known safe and effective treatments, or following advice from poorly trained health professionals in an unregulated environment [43]. Findings from studies such as ours can inform regulatory and policy frameworks, the designing of public health messages and the nature of provider-patient communication regarding T&CM use; all geared towards ensuring safe and informed care for Ebola survivors.

Our study results also indicate that Ebola survivors who use T&CM were concerned more with their personal experience of T&CM effectiveness than with clinical evidence, which is congruent with findings from studies of T&CM use among cancer survivors [44,45] and pregnant women [27]. The absence of clinical evidence of effectiveness and safety of the commonly used T&CM in the general population and among Ebola survivors in Sierra Leone, and coupled with the notion that T&CM is inherently safe and effective may help explain their preference for personal experience of T&CM over clinical evidence. While few preclinical efficacy [46,47] and toxicity [48] studies of some medicinal plants considered traditional medicine have been conducted in Sierra Leone, clinical research examining the safety and efficacy of commonly used T&CM especially herbal medicines in Sierra Leone is lacking. The availability of scientific evidence of T&CM effectiveness and safety is important in ensuring that it is effectively used. T&CM users are known to assume that T&CM is safe and effective [4] and that their decision to use T&CM is informed by non-professionals sources [42]. Therefore, it is imperative that robust efficacy and safety studies (preclinical, clinical and post-market surveillance) are conducted on commonly used T&CM in Sierra Leone that will inform personal and clinical decision making with regards T&CM.

Our study finding that survivors who are T&CM users want more control over their health is in line with insights from the current post-infectious sequelae literature both within [4,49] and outside [25] of Africa, including those among survivors of severe acute respiratory syndrome (SARS) in Hong Kong [50]. The sense of fear, vulnerability and perceived loss of control over their health due to the physical, psychosocial and economic problems experienced by Ebola survivors [18,51,52] may be a source of attraction toward T&CM. This may be particularly in view of the failures of conventional health options to address these needs, which allows survivors to have the sense of control, autonomy and active participation in decisions regarding their health and wellbeing [24,25,26,49].

T&CM is perceived to allow patients to take ownership of their health and well-being by allowing them to actively seek information and make decisions about treatment modalities that prove to be beneficial without being instructed on what to do [26,53]. Ebola survivors’ sense of control on one hand may affect their willingness to disclose their T&CM use status to their healthcare provider as disclosure of T&CM may be perceived by patients as shifting the power to their healthcare provider to make health decisions for them [53]. On the other hand, the sense of control that T&CM provides may also make survivors more assertive and therefore more communicative with their healthcare provider [53]. Notwithstanding, it is important that healthcare providers proactively seek to ask about survivors’ possible T&CM use as decisions regarding the efficacy and safety of T&CM often fail to be informed by reliable information sources [42], and such use may be indicative of unmet needs.

The majority of T&CM users and non-users amongst the Ebola survivors in our study agreed that medical doctors should be able to advise their patients about T&CM. Although this is in contrast to some studies in other conditions and in other countries [27], our finding suggests that Ebola survivors in Sierra Leone regardless of their T&CM use status will prefer to get advice about T&CM from a conventional medicine provider. The expectation of Ebola survivors for conventional medicine practitioners to be able to provide advice on T&CM illustrates a potential need for healthcare providers to routinely initiate and incorporate discussions about T&CM during consultation with Ebola survivors. The provision of evidence-based information on T&CM to patients requires healthcare providers to be knowledgeable about the safety and efficacy of commonly used T&CM among Ebola survivors.

### Study Limitations

One limitation of our study is that the data collected is self-reported, which may reflect recall bias. Moreover, our findings are not necessarily representative of the perceptions and experiences of Ebola survivors in other countries. Also, due to the cross-sectional nature of our study, a cause-effect relationship cannot be deduced from our results. In addition, we excluded Ebola survivors in our study with conditions that limit their ability to accurately provide information or participate in the study and may put the research and other participants at risk. Although the demographic characteristics of Ebola survivors excluded are similar to those included in our study, those excluded may have held different views with regards to the attitude statements considered in our study.

## 5. Conclusions

Findings from our study have provided insights into the reasons for T&CM use among Ebola survivors in Sierra Leone. Ebola survivors who are T&CM users value safety, personal experience of effectiveness, patient autonomy and the need to boost the body’s immunity when using T&CM. Such reasons for T&CM use among Ebola survivors are useful to conventional health providers, in improving practitioner-patient communication regarding T&CM and identify survivors’ beliefs about T&CM that might be targets for public education among Ebola survivors by policymakers and health providers.

## Figures and Tables

**Table 1:** Socio-demographic and health related characteristics of Ebola survivors (n = 358).

Characteristics	Variables	Total
		n (%)
Age Group	18–33 years	194 (54.2)
	34–49 years	134 (37.4)
	≥50 years	30 (8.4)
Sex	Male	135 (37.7)
	female	223 (62.3)
Educational Status	Non-formal education	147 (41.1)
	Primary	44 (12.3)
	Secondary	126 (35.2)
	Tertiary	41 (11.5)
Religious Affiliation	Christianity	92 (25.7)
	Islam	266 (74.3)
Marital Status	Single	100 (27.9)
	Married/Cohabitating	171 (47.8)
	Divorced/Separated/widowed	87 (24.3)
Monthly Income (Leones)	Less than 500,000	252 (70.4)
	500,000–1million	94 (26.3)
	>1million	12 (3.4)
Residential Area	Urban	219 (61.2)
	Rural	139 (38.8)
Region	Northern region (Bambali district)	120 (33.5)
	Southern region (Bo district)	55 (15.4)
	Eastern region (Kenema district)	62 (17.3)
	Western Area	121 (33.8)
Current Perceived Health Status	Very good/Good	96 (26.8)
	Fair/poor	262 (73.2)
Duration (Months) since Discharged from ETC	≤3 years	27 (7.5)
	>3 years	331 (92.5)
Known Chronic Disease	Yes	46 (12.8)
	No	312 (87.2)

**Table 2:** Traditional and complementary healthcare (T&CM) utilization among Ebola survivors (n = 358).

T&CM Utilization	Variable	n (%)
	Users	163 (45.5)
	Non-user	195 (54.5)
Concurrent use of both conventional medicine and T&CM	Yes	62 (17.3)
	No	296 (82.7)
Types of T&CM use—multiple choice	Herbal medicine	136 (83.4)
	Animal Extract	23 (14.1)
	Prayer/faith healing	60 (36.8)
	Acupuncture	1 (0.6%)
	Massage	33 (20.2)
	Others (scarification local surgery)	10 (6.1)
Average monthly direct cost of T&CM per person		= Le 14,036 approximately 1.8 US dollars (0 to 10.9 USD)

**Table 3:** Attitude of Ebola survivors towards the use of T&CM.

Attitude of Ebola Survivors towards the Use of T&CM	Agree	Neutral	Disagree
	n (%)	n (%)	n (%)
T&CM is a more natural than conventional medicine	257 (71.8)	20 (5.6)	81 (22.6)
TCAM boosts my immune system/resistance	114 (31.8)	44 (12.3)	200 (55.9)
T&CM has fewer side-effects than conventional medicine	82 (22.9)	58 (16.2)	218 (60.9)
T&CM promotes a holistic approach to health	100 (27.9)	55 (15.4)	203 (56.7)
T&CM gives me more control over my health/body	120 (33.5)	78 (21.8)	160 (44.7)
Knowledge about the evidence of T&CM effectiveness is important to me as a patient	213 (59.5)	25 (7.0)	120 (33.5)
My personal experience of the effectiveness of T&CM is more important than clinical evidence	163 (45.5)	36 (10.1)	159 (44.4)
T&CM is a better preventative measure than conventional medicine	58 (16.2)	50 (14.0)	250 (69.8)
T&CM needs to be tested for safety/side-effects	268 (74.9)	27 (7.5)	63 (17.6)
T&CM practitioner spends a longer time with patients in consultations compared with a medical doctor	177 (49.4)	47 (13.1)	134 (37.4)
T&CM practitioner provides more support to his/her patient than a medical doctor	73 (20.4)	47 (13.1)	238 (66.5)
I find it easier to talk to a T&CM practitioner than a medical doctor	100 (27.9)	33 (9.2)	225 (62.8)
Medical doctors should be able to advise patient about T&CM	313 (87.4)	16 (4.5)	29 (8.1)

**Table 4:** Comparison of attitudes between users and non-users of TCAM treatment among Ebola survivors.

Attitude Statements	Variables	T&CM USE		
		Users	Non-User	*p*-Value
		n (%)	n (%)	
T&CM is a more natural than conventional medicine	Disagree	10 (6.1)	71 (36.4)	<0.001
	Neutral	6 (3.7)	14 (7.2)	
	Agree	147 (90.2)	110 (56.4)	
T&CM boosts my immune system/resistance	Disagree	46 (28.2)	154 (79.0)	<0.001
	Neutral	14 (8.6)	30 (15.4)	
	Agree	103 (63.2)	11 (5.6)	
T&CM has fewer side-effects than conventional medicine	Disagree	69 (42.3)	149 (76.4)	<0.001
	Neutral	30 (18.4)	28 (14.4)	
	Agree	64 (39.3)	18 (9.2)	
T&CM promotes a holistic approach to health	Disagree	60 (36.8)	143 (73.3)	<0.001
	Neutral	23 (14.1)	32 (16.4)	
	Agree	80 (49.1)	20 (10.3)	
T&CM gives me more control over my health/body	Disagree	39 (23.9)	121 (62.1)	<0.001
	Neutral	28 (17.2)	50 (25.6)	
	Agree	96 (58.9)	24 (12.3)	
Knowledge about the evidence of T&CM is important to me as a patient	Disagree	18 (11.0)	102 (52.3)	<0.001
	Neutral	3 (1.8)	22 (11.3)	
	Agree	142 (87.1)	71 (36.4)	
My personal experience of the effectiveness of T&CM is more important than clinical evidence	Disagree	19 (11.7)	140 (71.8)	<0.001
	Neutral	11 (6.7)	25 (12.8)	
	Agree	133 (81.6)	30 (15.4)	
T&CM is a better preventative measure than conventional medicine	Disagree	87 (53.4)	163 (83.6)	<0.001
	Neutral	24 (14.7)	26 (13.3)	
	Agree	52 (31.9)	6 (3.1)	
T&CM needs to be tested for safety/side-effects	Disagree	30 (18.4)	33 (16.9)	0.409
	Neutral	9 (5.5)	18 (9.2)	
	Agree	124 (76.1)	144 (73.8)	
T&CM practitioner spends a longer time with patients in consultations compared with a medical doctor	Disagree	59 (36.2)	75 (38.5)	0.257
	Neutral	17 (10.4)	30 (15.4)	
	Agree	87 (53.4)	90 (46.2)	
T&CM practitioner provides more support to his/her patient than a medical doctor	Disagree	87 (53.4)	151 (77.4)	<0.001
	Neutral	20 (12.3)	27 (13.8)	
	Agree	56 (34.4)	17 (8.7)	
I find it easier to talk to a T&CM practitioner than a medical doctor	Disagree	78 (47.9)	147 (75.4)	<0.001
	Neutral	13 (8.0)	20 (10.3)	
	Agree	72 (44.2)	28 (14.4)	
Medical doctors should be able to advise patient about T&CM	Disagree	13 (8.0)	16 (8.2)	0.496
	Neutral	5 (3.1)	11 (5.6)	
	Agree	145 (89.0)	168 (86.2)	

**Table 5:** Logistic regression analyses demonstrating attitudes towards T&CM and use of TCAM treatment among Ebola survivors.

Attitude Statements	Variables	T&CM Treatment Use		
		Adjusted OR	95% C.I.	*p*-Value
T&CM boosts my immune system/resistance	Disagree	1		
	Neutral	0.83	0.29–2.39	0.729
	Agree	3.89	1.57–9.63	0.003
T&CM has fewer side-effects than conventional medicine	Disagree	1		
	Neutral	1.92	0.73–5.08	0.189
	Agree	5.03	1.92–13.19	0.001
T&CM gives me more control over my health/body	Disagree	1		
	Neutral	1.68	0.68–4.20	0.263
	Agree	4.15	1.74–9.89	0.001
My personal experience of the effectiveness of T&CM is more important than clinical evidence	Disagree	1		
	Neutral	2.07	0.58–7.41	0.263
	Agree	13.72	6.10–30.84	<0.001

Note: C.I. =Confidence Interval; OR= Odd ratio.
